# Doping Attitudes, Beliefs, and Practices among Young, Amateur Croatian Athletes

**DOI:** 10.3390/sports9020025

**Published:** 2021-02-09

**Authors:** Ivan Miskulin, Danijela Stimac Grbic, Maja Miskulin

**Affiliations:** 1 Department of Public Health, Faculty of Medicine Osijek, Josip Juraj Strossmayer University of Osijek, 31000 Osijek, Croatia; maja.miskulin@mefos.hr; 2 Department of Social Medicine and Organization of Health Care, Andrija Stampar School of Public Health, School of Medicine, University of Zagreb, 10000 Zagreb, Croatia; danijela.stimac@stampar.hr

**Keywords:** performance enhancing drugs, athletes, attitude, prevention, Croatia

## Abstract

Recent studies revealed that amateur athletes, especially young ones, have an increasing tendency of performance-enhancing drugs (PEDs) usage. The aim of this study was to explore PEDs attitudes, beliefs, and practices among young, amateur Croatian athletes. This cross-sectional study using a specially designed questionnaire as a research tool was done during the August 2019 to January 2020 period among a convenient sample of 400 amateur athletes of median age 18 (interquartile range 15 to 21) years. The prevalence of current PEDs usage was 1.3%, while past PEDs usage prevalence was 3.3%. Current PEDs usage was more frequent among young adults (*p* = 0.048) and athletes playing individual sports (*p* = 0.001). Athletes who were engaged in sports from one to five years had more permissive attitudes toward PEDs (*p* < 0.001) as measured by the Performance Enhancement Attitude Scale. Female athletes had more positive beliefs about PEDs usage (*p* = 0.008). The study did not establish any correlation between current or past PEDs usage and attitudes toward PEDs as well as beliefs about PEDs usage. However, there was a weak positive correlation between attitudes toward PEDs and athletes’ beliefs about PEDs usage (r_s_ = 0.465, *p* < 0.001). PEDs usage is present among young Croatian amateur athletes. There is a need for interventions directed toward the prevention of PEDs usage in an observed subgroup of athletes.

## 1. Introduction

Physical activity is very important for mental and physical health and well-being in all age groups of people, especially young people [1,2,3,4]. However, the beneficial effects of physical activity can be diminished by certain behaviors such as the violation of anti-doping rules [1]. The World Anti-Doping Agency (WADA) has precisely defined what an anti-doping rule violation means. Consequently, anti-doping rule violations include the presence of a banned substance or its metabolites in athletes’ biological samples, the use or attempted use of banned substances or methods by athletes, the athletes’ avoidance or refusal of doping control, unauthorized interference or attempted unauthorized use of any part of the doping control by the athlete or other person, and the possession of a banned substance or method by the athlete or supporting person. Violations of these rules also include the smuggling or attempted smuggling of any banned substance or method by an athlete or other person as well as the application or attempted application to any athlete in competition of any banned substance or method by an athlete or other person. Finally, an anti-doping rule violation includes the application or attempted application to any athlete out of competition of any banned substance or method that is banned out of competition, complicity or attempted complicity in an anti-doping rule violation by the athlete or other person, banned association of athletes or other persons and activities of the athlete or other person aimed at discouraging or distracting from reporting a case of anti-doping rule violation to the competent authorities. All of this represents cheating in sports [5,6,7]. Today, doping usage is recognized as an important issue in sport where term “doping” generally indicates the use of illegal or prohibited performance enhancing substances e.g., drugs [8,9]. Studies dealing with the issue point to the fact that not only elite athletes use performance-enhancing drugs (PEDs) but also those who engage in amateur and recreational sports [10,11,12,13], sometimes to an even greater extent than professional athletes [8,14]. Following the latter reason, PEDs have been recognized as a rising public health problem globally [8,11,15]. In the context of the previously written, the fact that the increase in the PEDs usage is particularly detected among young people is particularly worrying [16,17,18].

Studies around the world had shown that from 0.6% to 5.0% of adolescents who practice sports use PEDs [19]. In a study from Italy, it was stated that 1.5% of 3498 Italian high school adolescents had used PEDs in previous three months [20]. Of particular concern is the fact that from 3.0% to 12.0% of adolescent athletes had used anabolic-androgenic steroids (AAS) at some point [11], which are the most commonly used illegal PEDs [21]. Recent analysis concerning the interconnection of age and the type of illegal PEDs usage is ambiguous, because some evidence emphasizes the adolescent age as riskier for AAS usage, while others show that AAS usage is more frequent among young adults [22]. In a study that included a sizeable number of young persons from five European countries (Germany, Italy, Greece, UK, and Cyprus) who practice amateur sports, the prevalence of current PEDs usage was 8.1%, while the prevalence of past PEDs usage was 10.2% [1]. The above-mentioned prevalence in young amateur athletes is certainly alarming, especially from the perspective of the adverse health effects that these substances have on young people’s health [23]. Namely, PEDs usage is associated with heart diseases, mental health disorders, diabetes, cancer, masculinization in females, and deficiency of naturally produced androgens in males [19,24,25,26]. Understanding why young amateur athletes tend to use PEDs contributes to evidence-based planning of antidoping preventive programs, because effective interventions need to target factors causally related to the PEDs usage [23]. As a result of that, it is highly important to understand and investigate factors that are potentially associated with the PEDs usage or factors with possible causal relationship with PEDs [27]. Related to this, recent studies showed that attitudes, beliefs, and perceived social norms can predict the intention of PEDs usage [28,29]. Regarding the attitudes, recent studies found a positive relationship between athletes’ attitudes toward PEDs and their intention to use them [30,31]. Considering the latter, the study of Barkoukis et al. showed that together with well-known predictors of doping intentions, which are attitudes and normative beliefs, beliefs about the causes of success in sports present another dimension of risk factor for PEDs susceptibility and usage [32]. In addition, studies have shown that belief that PEDs are used in a specific sport should be recognized as a risk factor associated with the use of these substances [33,34].

Following all the above and considering that there is a lack of studies dealing with the issue of PEDs usage in recreational athletes in Croatia, the aim of this study was to explore PEDs attitudes, beliefs, and practices among young, amateur Croatian athletes.

## 2. Materials and Methods

**Figure 1:**
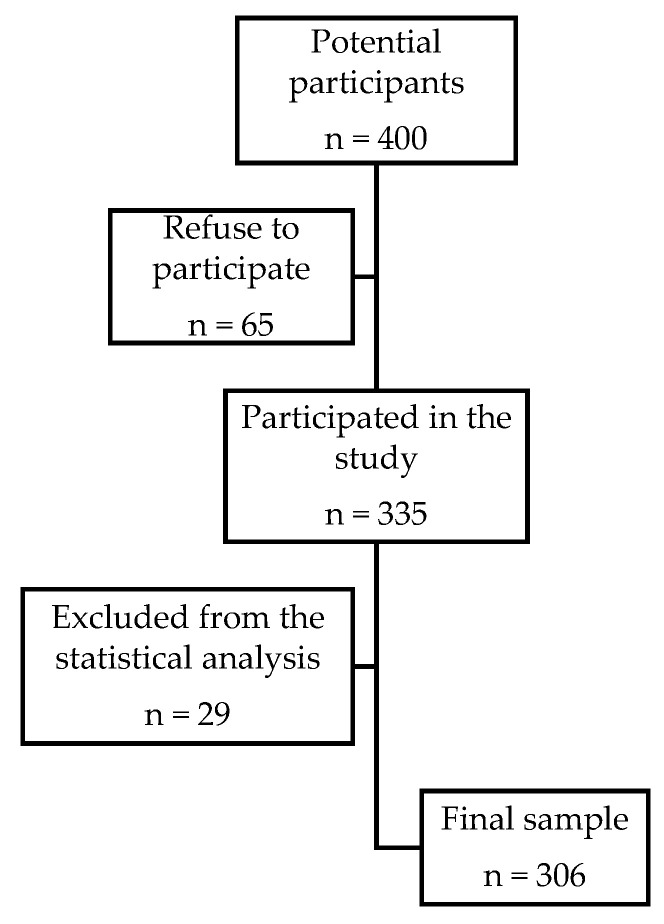
Participants’ recruitment process.

This cross-sectional study was conducted during the August 2019 to January 2020 period among a convenient sample of 400 amateur athletes from Osijek, Eastern Croatia. The amateur athlete is a person who practices sports solely for personal satisfaction without any monetary gain. The research was conducted at the Occupational and Sports Medicine Office in Osijek, and potential participants were enrolled in the study during their regular medical examinations by the occupational and sports medicine specialist. All potential participants received a written exposition of the study research protocol and its objectives and were asked to voluntarily participate in the proposed research by completing an anonymous questionnaire. The research was approved by the Ethics Committee of the Osijek Health Center (Ethical approval code: 03-530-20), and each participant gave his or her written consent to participate in the research before completing the anonymous questionnaire. A total of 400 potential participants were invited to participate in the research, and the overall response rate was 83.75% (335/400), since 65 participants refused to participate. Statistical analysis included 306 questionnaires that were fully completed, while 29 collected questionnaires were omitted from the statistical analysis because they were not fully completed (Figure 1).

The research tool of this study was a 25-item anonymous questionnaire: four questions on demographics (sex, age, the name of sport that participant practice, and the length of practicing sports in years); 17 questions regarding the general attitudes toward PEDs; two questions regarding participants’ PEDs beliefs; and two questions regarding participants’ current and past PEDs behavior.

Data on study participants’ demographics were stratified in categories for the purpose of statistical analysis. All participants were classified in two groups according to age: adolescents (aged 13 to 18 years) and young adults (aged 19 to 24 years). All sports were further categorized in two categories as follows: individual sports (sports in which participants practice as individuals) and team sports (sports that involve athletes working together as a team). The length of practicing sports in years was also categorized in two categories as follows: one to five years and six or more years of sports engagement.

Attitude toward PEDs was defined as an individual’s predisposition toward the use of banned performance-enhancing substances and methods and was quantitatively measured by the Performance Enhancement Attitude Scale (PEAS), which was proposed by Petróczi [35]. The PEAS comprised of 17 items on a six-point Likert-type scale (strongly disagree (1), strongly agree (6), and no neutral, middle point), and all of them were scored in the same direction. The overall score ranged between 17 and 102 [36]. A high score reflects a permissive attitude toward PEDs, while a low score indicates an intolerant attitude [35]. Even though some recent studies proposed the usage of the 11-item, 8-item, and 6-item versions of PEAS [37,38,39], in this study, we used the original 17-item version of the tool that was previously adapted to Croatian and used in the Croatian population [5]. Another reason for such a decision, besides the fact that the original 17-item version is the most extensively used tool for the assessment of PEDs attitudes among adult and adolescent athletes, is the finding of the study done by Nicholls et al. who concluded that the 8-item version of the PEAS demonstrated the best fit for adults, but no model exhibited a good fit with adolescent athletes [40].

The PEDs usage beliefs of study participants were assessed with two questions regarding PEDs usage and other methods for enhancement of sports results allowed to either elite or all athletes as proposed by Petróczi [35]. Each question had three possible answers scored as follows: “yes, without limitations”—2 points, “yes, with limitations”—1 point, and “absolutely no”—0 points. The higher score marked higher probability of beliefs that PEDs should be allowed.

The PEDs behavior of study participants was assessed with two questions regarding the current use and past experience with performance-enhancing substances as proposed by Petróczi [35]. Each question had four possible answers scored as follows: “yes”—3 points; “yes—but only for the purpose of treatment”—2 points; “no”—0 points; and “I don’t want to answer”—1 point. In further statistical analysis, answers “yes”, “yes—but only for the purpose of treatment”, and “I don’t want to answer” were considered as a sign of PEDs usage.

Participants needed around 10 min for completing the entire questionnaire. After completing the questionnaire, they we asked to put their completed questionnaires in a specially designed box that was located in the waiting room of the occupational medicine and sports office. The box for the collection of completed questionnaires could not be opened or seen through.

The normality of data distributions was checked by the Kolmogorov–Smirnov test. Descriptive statistics was used to describe the basic features of the data in a study. The categorical variables were described in absolute and relative frequencies. For the description of the numerical variables, we used median and interquartile range. The Mann–Whitney U test was applied for the comparison of numerical variables. Fisher’s exact test was applied for the comparison of categorical variables. Spearman’s correlation was applied to test the correlation between current and past PEDs usage, and attitudes toward PEDs measured by PEAS scale and also correlation between current and past PEDs usage and PEDs usage beliefs as well as correlation between attitudes toward PEDs measured by PEAS scale and PEDs usage beliefs of study participants. Statistical software Statistica for Windows 2010 (version 10.0, StatSoft Inc., Tulsa, OK, USA) was used for data analyses. On all statistical analyses, two-sided *p*-values of 0.05 were considered significant.

## 3. Results

### 3.1. Amateur Athletes’ Characteristics

Participants’ median age was 18.0 years (interquartile range 15.0–21.0); 62.7% were females and 52.9% were adolescents (aged 13–18 years). According to the sports they played, there were 26.8% of those who played volleyball, 26.1% of those who played football, 15.7% of those who played handball, 10.5% of those who played basketball, and 20.9% of those who were engaged in other sports. Among all athletes, 80.4% played team sports, and according to the length of sport engagement, 50.3% were engaged in a particular sport for six or more years (Table 1).

### 3.2. Athletes’ Attitudes toward PEDs Usage According to the PEAS Scale

The median value of all athletes’ general attitudes toward PEDs usage according to the PEAS scale was 29.00 (interquartile range from 22.75 to 42.25). There was no statistically significant difference between male and female athletes according to their general attitudes toward PEDs usage measured by the PEAS scale (Mann–Whitney U test; *p* = 0.099). In addition, there was no statistically significant difference between athletes’ general attitudes toward PEDs usage according to their age group (Mann–Whitney U test; *p* = 0.145) and according to the type of sports they played (Mann–Whitney U test; *p* = 0.405). The study revealed that there was a statistically significant difference between athletes’ general attitudes toward PEDs usage according to the length of their sport engagement (Mann–Whitney U test; *p* < 0.001) (Table 2).

### 3.3. Athletes’ PEDs Usage Beliefs

The median value of all athletes’ PEDs usage beliefs was 0.00 (interquartile range from 0.00 to 1.00). The study showed that there was a statistically significant difference in PEDs usage beliefs between male and female athletes (Mann–Whitney U test; *p* = 0.008). There was no statistically significant difference in PEDs usage beliefs between athletes according to their age group (Mann–Whitney U test; *p* = 0.346); according to the type of sports they played (Mann–Whitney U test; *p* = 0.522); and according to the length of their sport engagement (Mann–Whitney U test; *p* = 0.663) (Table 3).

### 3.4. Athletes’ PEDs Usage Practices

Considering the current PEDs usage, 1.3% of athletes did not want to answer this question, which was considered as a positive PEDs practice. Regarding past PEDs usage, 2.6% of athletes did not want to answer this question, and 0.7% of athletes admitted that they had used PEDs but only for the purpose of treatment, yielding 3.3% of athletes with a past positive PEDs practice. The current PEDs usage was more frequent among athletes belonging to the young adult age group (Fisher’s exact test; *p* = 0.048) and athletes who played individual sports (Fisher’s exact test; *p* = 0.001), while there were no statistically significant differences in the frequency of current PEDs usage according to the athletes’ sex and the length of their sport engagement (Fisher’s exact test; *p* = 0.301 and *p* = 0.060, respectively). The past PEDs usage was more frequent among athletes who played individual sports (Fisher’s exact test; *p* = 0.005), while there were no statistically significant differences in the frequency of past PEDs usage according to the athletes’ sex, age, group and the length of their sport engagement (Fisher’s exact test; *p* = 0.183, *p* = 0.754 and *p* = 0.092, respectively) (Table 4).

### 3.5. Interconnections between Athletes’ PEDs Attitudes, Beliefs, and Practices

Our study showed that there was no correlation between athletes’ attitudes toward PEDs usage measured by the PEAS scale and their current and past PEDs usage practices (r_s_ = 0.162, *p* = 0.005 and r_s_ = 0.234, *p* < 0.001, respectively). It further revealed that there was no correlation between athletes’ PEDs usage beliefs and their current and past PEDs usage practices (r _s_ = 0.220, *p* < 0.001 and r_s_ = 0.236, *p* < 0.001, respectively). Finally, our study showed that there was a weak positive correlation between athletes’ attitudes toward PEDs usage measured by the PEAS scale and their PEDs usage beliefs (r_s_ = 0.465; *p* < 0.001).

## 4. Discussion

The present study confirmed that PEDs usage is an important issue for amateur athletes in Croatia since it has been established that the prevalence of current PEDs usage in the observed population of athletes was 1.3%, while the prevalence of past PEDs usage among them was 3.3%. Established prevalences are lower than those found in a study comprised of a sizeable number of young persons from five European countries who exercise and practice amateur sports where the prevalence of current PEDs usage was 8.1% and past PEDs usage was 10.2% [1]. The prevalence of the current PEDs usage of 1.3% established in this study is almost identical to the established current PEDs usage prevalence of 1.5% in the study from Italy [20]. The explanation for these results is probably associated with the mean age of study sample participants. Namely, in the study that included five European countries, the mean age of participants was 21.6 years [1], and in the study from Italy, it was 16.5 years [19], while in this study, it was 18.0 years, and there is some evidence that PEDs usage is more frequent among young adults than among adolescents [22]. This study confirmed the previously mentioned fact that amateur athletes from the young adults’ group had used PEDs more frequently in comparison to the adolescents. Unlike the other studies, this study did not determine the statistically significant difference in a frequency of PEDs usage among male and female athletes [16,19]. The present study revealed that the current and the past PEDs usage prevalence was higher among amateur athletes who played individual sports, which is in concordance with the finding of a study done by Aguilar-Navarro et al., who concluded that the incidence of PEDs usage was not uniform in all sports disciplines, suggesting that some specific sports might present a greater use of banned substances [41].

The median value of young Croatian amateur athletes’ general attitudes toward PEDs usage according to the PEAS scale was 29.00, which was lower than the PEAS score of 35.05 identified in a group of amateur football players from a Spain, suggesting more restrictive attitudes of Croatian athletes toward PEDs [42]. Similar to some other studies, this study did not show differences in general attitudes toward PEDs usage between males and females [36,43]. Unlike the study from Korea, this study did not show statistically significant differences in general attitudes toward PEDs usage between athletes playing individual and team sports [36]. Similar to study in Kenya, this study also did not show differences in athletes’ general attitudes toward PEDs usage between young adults and adolescents [44]. Finally, this study established statistically significant difference in athletes’ general attitudes toward PEDs regarding the length of sport engagement, where athletes who were engaged in sports for one to five years had more permissive attitudes toward PEDs usage in comparison with the athletes engaged in sports for six or more years. This can be explained by the fact that the athletes who play sports for a shorter period need to be more competitive than athletes who played it longer [45].

Furthermore, this study showed that female athletes scored higher in questions regarding PEDs usage beliefs that made them more prone to beliefs that PEDs should be allowed. The latter finding is opposite to the studies of Tavares et al. and Backhouse et al., who stated that male athletes scored higher regarding beliefs that PEDs should be allowed [28,46]. Other observed variables did not show statistically significant differences regarding PEDs usage beliefs, but these findings should be further investigated in a larger sample.

Considering the interconnections between the athletes’ PEDs attitudes, beliefs, and practices, this study did not show correlation between athletes’ attitudes toward PEDs usage measured by PEAS scale and their current or past PEDs usage practices, although the PEDs attitude is the key variable in predicting athlete’s intention of PEDs usage [47]. The latter finding contrasts with the study of Muwonge et al., who showed that the highest PEAS score was observed in athletes who have had a PEDs experience [43]. Even though the recent study by Tavares et al. indicated that beliefs could predict the intention to use PEDs, this study did not show such a connection [28]. Finally, this study showed a positive interconnection between athletes’ attitudes toward PEDs usage measured by the PEAS scale and their PEDs usage beliefs. The theory of reasoned action suggests that attitudes are influenced by beliefs, and because of that, athletes who have strong positive beliefs about the effectiveness of PEDs are expected to have positive attitudes toward PEDs [48]. Furthermore, Petróczi reported that stronger beliefs about PEDs were associated with more permissive PEDs attitudes, while Chan et al. showed that beliefs about PEDs usage advantages positively predicted PEDs attitudes [35,49].

While providing insightful findings about PEDs usage in young amateur Croatian athletes, the present study was not without limitations. Firstly, results depend on self-reported data and thus were potentially affected by reporting and social desirability biases. Secondly, the prevalence of current and past PEDs usage reported in this study may actually be under-estimated. In addition, this study did not explore other possible factors that can contribute to PEDs usage, such as, for example, the usage of nutritional supplements, which is important because a previous study revealed that nutritional supplements users have a tendency toward more permissive PEDs usage attitudes and are more prone to use PEDs themselves [50]. Future studies may additionally refer to mentioned limitations and ensure more detailed insight into the PEDs usage issues in amateur sports.

## 5. Conclusions

The present study revealed a worrying prevalence of current and past PEDs usage among young Croatian amateur athletes. Such a finding suggests a great need for the development of interventions directed toward the prevention of PEDs usage in the observed population subgroup. In order to prepare evidence based and highly effective prevention programs, a better understanding of potential correlates and determinants of PEDs usage are needed.

## Figures and Tables

**Table 1:** Sociodemographic factors and sports-associated factors.

Sociodemographic and Sports-Associated Factors	N	%
Sex		
Male	114	37.3
Female	192	62.7
Age group (years)		
Adolescents (13–18)	162	52.9
Young adults (19–24)	144	47.1
Athletes’ sport		
Volleyball	82	26.8
Football	80	26.1
Handball	48	15.7
Basketball	32	10.5
Other sports	64	20.9
Type of sport		
Individual	60	19.6
Team	246	80.4
Length of sport engagement		
1–5 years	152	49.7
≥6 years	154	50.3

**Table 2:** Athletes’ general attitudes toward performance-enhancing drugs (PEDs) measured by the Performance Enhancement Attitude Scale (PEAS) scale according to their length of sport engagement.

Sociodemographic and Sports-Associated Factors		Athletes’ General Attitudes toward PEDs	*p* *
		Median (Q1–Q3)	
Sex	Male	30.00 (24.00–42.00)	0.099
	Female	27.50 (22.00–43.00)	
Age group	Adolescents	31.00 (24.00–42.00)	0.145
	Young adults	26.00 (21.50–43.50)	
Type of sport	Individual	25.00 (22.00–42.00)	0.405
	Team	30.00 (23.00–43.00)	
Length of sport engagement	1–5 years	34.50 (24.50–44.00)	<0.001
	≥6 years	25.00 (22.00–37.00)	

* Mann–Whitney U test.

**Table 3:** Athletes’ PEDs usage beliefs according to their sex.

Sociodemographic and Sports-Associated Factors		Athletes’ PEDs Usage Beliefs	*p* *
		Median (Q1–Q3)	
Sex	Male	0.00 (0.00–0.00)	0.008
	Female	0.00 (0.00–1.00)	
Age group	Adolescents	0.00 (0.00–1.00)	0.346
	Young adults	0.00 (0.00–1.00)	
Type of sport	Individual	0.00 (0.00–1.00)	0.522
	Team	0.00 (0.00–1.00)	
Length of sport engagement	1–5 years	0.00 (0.00–1.00)	0.663
	≥6 years	0.00 (0.00–1.00)	

* Mann–Whitney U test.

**Table 4:** Current and past PEDs usage and associated factors.

Factors	Current PEDs Usage (Yes/No)	Past PEDs Usage (Yes/No)
Sociodemographic		
Sex	*p* = 0.301 *	*p* = 0.183 *
Age group	*p* = 0.048 *	*p* = 0.754 *
Sports associated		
Type of sport	*p* = 0.001 *	*p* = 0.005 *
The length of sport engagement	*p* = 0.060 *	*p* = 0.092 *

* Fisher’s exact test.

## Data Availability

The data presented in this article are available on request from the corresponding author.
